# Development of the multiplex imaging chamber at PAL-XFEL

**DOI:** 10.1107/S1600577524001218

**Published:** 2024-03-22

**Authors:** Junha Hwang, Sejin Kim, Sung Yun Lee, Eunyoung Park, Jaeyong Shin, Jae Hyuk Lee, Myong-jin Kim, Seonghan Kim, Sang-Youn Park, Dogeun Jang, Intae Eom, Sangsoo Kim, Changyong Song, Kyung Sook Kim, Daewoong Nam

**Affiliations:** a Department of Physics, Pohang University of Science and Technology, Pohang 37673, Republic of Korea; b Photon Science Center, Pohang University of Science and Technology, Pohang 37673, Republic of Korea; c Center for Ultrafast Science on Quantum Matter, Max Planck POSTECH/Korea Research Initiative, Pohang 37673, Republic of Korea; d XFEL Beamline Department, Pohang Accelerator Laboratory, Pohang University of Science and Technology, Pohang 37673, Republic of Korea; University of Tokyo, Japan

**Keywords:** coherent diffraction imaging, wide-angle X-ray diffraction, X-ray emission spectroscopy, XFELs, ultrafast dynamics

## Abstract

Various X-ray techniques are employed to investigate specimens in diverse fields. Generally, scattering and absorption/emission processes occur due to the interaction of X-rays with matter. The output signals from these processes contain structural information and the electronic structure of specimens, respectively. The combination of complementary X-ray techniques improves the understanding of complex systems holistically. In this context, we introduce a multiplex imaging instrument that can collect small-/wide-angle X-ray diffraction and X-ray emission spectra simultaneously to investigate morphological information with nanoscale resolution, crystal arrangement at the atomic scale and the electronic structure of specimens.

## Abstract 2

A new instrument that can collect small-/wide-angle X-ray diffraction and X-ray emission spectra simultaneously to probe the physical and chemical structures of specimens is introduced.

## 1. Introduction

X-ray free-electron lasers (XFELs) generate X-ray pulses with very short duration, typically on the order of a few tens of femtoseconds (Emma *et al.*, 2010 ▸; Ishikawa *et al.*, 2012 ▸; Kang *et al.*, 2017 ▸; Decking *et al.*, 2020 ▸; Prat *et al.*, 2020 ▸). This prominent property makes XFELs well suited for exploring ultrafast phenomena in combination with a pump source, *e.g.* femtosecond optical lasers (Behrens *et al.*, 2014 ▸; Kim *et al.*, 2019 ▸). Time-resolved X-ray experiments with XFELs are widely employed in the study of ultrafast phenomena using the pump–probe method (Schmidt, 2013 ▸; Gerber *et al.*, 2015 ▸; Kang *et al.*, 2020 ▸).

Photoinduced dynamics are of keen interest in various fields (Canton *et al.*, 2015 ▸; Kern *et al.*, 2013 ▸, Kim *et al.*, 2015 ▸; Aquila *et al.*, 2012 ▸; Tenboer *et al.*, 2014 ▸; Nango *et al.*, 2016 ▸; Assefa *et al.*, 2020 ▸). Electrons absorb the photons from the optical pump laser. The excessive energy of electrons is subsequently transferred to the lattice through electron–phonon coupling. This perturbs the atomic arrangement of the sample. This transient energy transfer process leads to a photoinduced phase transition. Rarely, a hidden or metastable state is discovered during this process, and it can unveil unexpected properties of specimens (Ichikawa *et al.*, 2011 ▸; Jung *et al.*, 2021 ▸; Mitrano *et al.*, 2016 ▸). Ultimately, investigating these processes allows a concrete understanding of the photoinduced dynamics of a specimen.

Coherent diffraction imaging (CDI) allows the user to obtain the electron density distribution of specimens at nanoscale resolution without using any X-ray lenses (Miao *et al.*, 2015 ▸). Coherent X-ray pulses generated from XFELs have been used to unveil high-resolution structural information of various specimens through single X-ray pulse exposure (Seibert *et al.*, 2011 ▸; Loh *et al.*, 2012 ▸; Xu *et al.*, 2014 ▸; Hantke *et al.*, 2014 ▸; Kimura *et al.*, 2014 ▸; van der Schot *et al.*, 2015 ▸; Ekeberg *et al.*, 2015 ▸; Cho *et al.*, 2021 ▸). Furthermore, morphological changes in specimens with nanometre to picosecond spatial–temporal resolution can be observed through pump–probe CDI (PPCDI) at XFELs (Clark *et al.*, 2013 ▸; Ihm *et al.*, 2019 ▸; Jung *et al.*, 2021 ▸; Shin *et al.*, 2023 ▸). Because X-ray microscopy provides structural information of the specimens intuitively, high-resolution imaging helps us to get a picture to understand ultrafast phenomena.

PPCDI can be extended to investigate irreversible processes for homogeneous specimens (Ihm *et al.*, 2019 ▸; Jung *et al.*, 2021 ▸; Shin *et al.*, 2023 ▸). However, nanoscale spatial resolution alone is not sufficient to fully understand photoinduced dynamics because early changes in these dynamics are observed at the atomic scale, such as lattice vibration. For a comprehensive understanding of ultrafast phenomena, complementary methods are needed to detect changes at the atomic scale. Recently, the simultaneous collection of small-/wide-angle diffraction signals has been demonstrated to track ultrafast phenomena (Shin *et al.*, 2023 ▸). Like this approach, collecting complementary information simultaneously using different methods is referred to as a multiplexing experiment. It allows experimental goals to be achieved in a time-saving and highly efficient manner. As the beam time at the XFEL is limited, the multiplexing experiment at the XFEL makes more efficient use of the XFEL beam time, and the high brightness of the XFEL enables single-shot observation with a single X-ray pulse.

In this paper, we introduce a multiplex imaging chamber designed to simultaneously collect small-/wide-angle diffraction signals and X-ray emission spectra from a sample. The purpose of this new instrument is to provide complementary information by collecting different signals, enabling a comprehensive understanding of specimens. Finally, we present commissioning results to show the performance of this instrument.

## 2. Multiplex imaging chamber

**Figure 1:**
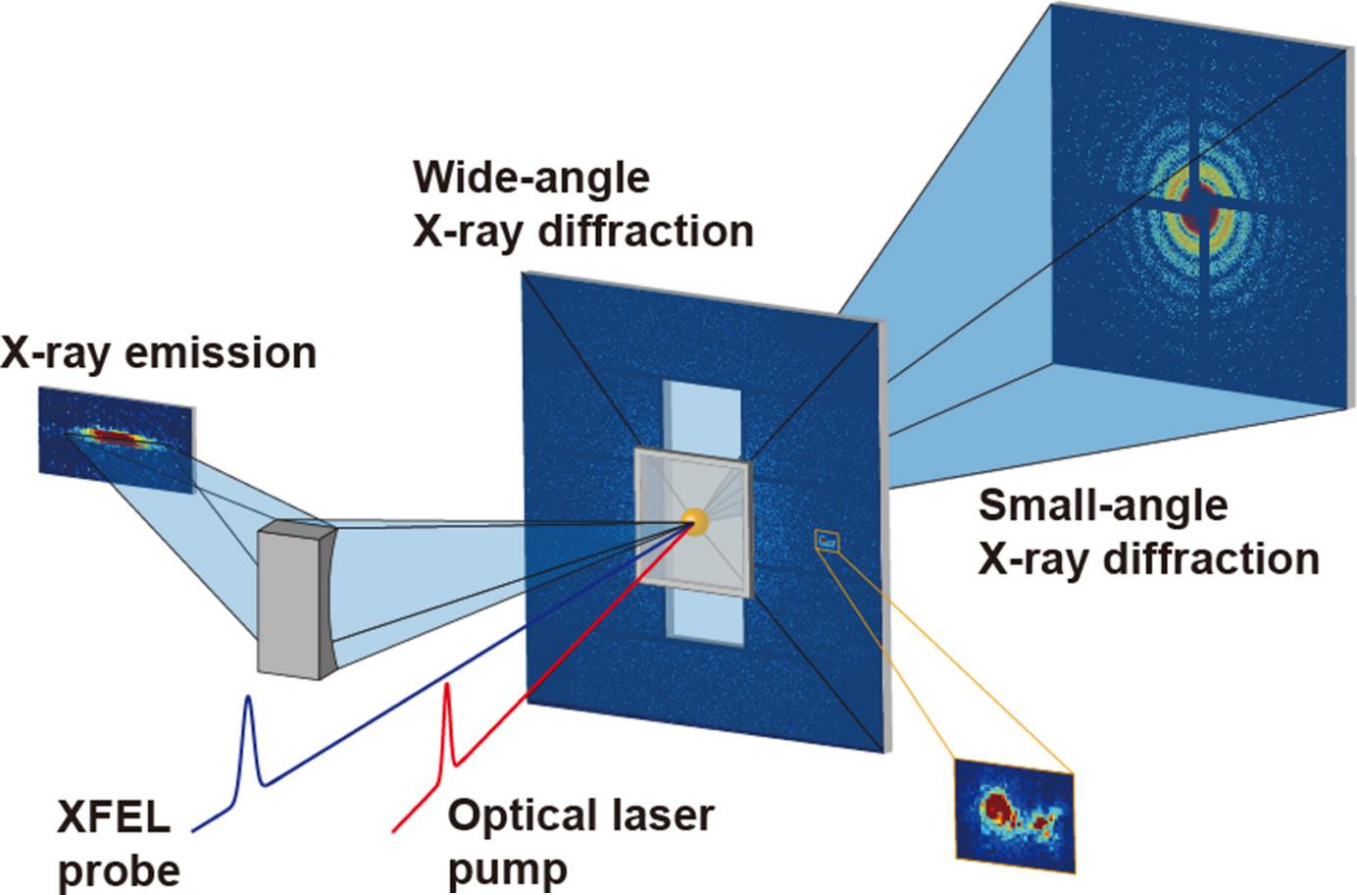
Schematic of the multiplex imaging experiment. It enables collection of small-/wide-angle X-ray diffraction and X-ray emission spectra simultaneously to investigate morphological information with nanoscale resolution, crystal arrangement at the atomic scale and the electronic structure of specimens.

After the successful lasing of the Pohang Accelerator Laboratory X-ray free-electron laser (PAL-XFEL) in 2016, an instrument designed for CDI experiments was introduced for early user experiments. The first version of the imaging instrument was equipped with a multiport charge-coupled device detector (Kameshima *et al.*, 2014 ▸) and operated at a frequency of 30 Hz to facilitate the examination of specimens’ structural information (Sung *et al.*, 2021 ▸). Later, the JUNGFRAU detector was introduced for user experiments (Redford *et al.*, 2018 ▸), enabling data collection with 60 diffraction patterns per second. Following this, the chamber exit port was modified to accommodate two additional JUNGFRAU 0.5 Megapixel (0.5M) detectors to collect Bragg peaks at a fixed 2θ angle of 30°. Notably, a multiplexing experiment was successfully demonstrated. This instrument allows the simultaneous collection of small-/wide-angle X-ray diffraction to investigate ultrafast melting phenomena (Shin *et al.*, 2023 ▸). However, the azimuthal coverage of these two detectors remains partial. Although this setup suffices for comprehending the atomic arrangement of polycrystalline samples, its effectiveness is limited when studying single-crystal samples because of the random distribution of sample orientations. A new instrument has thus been developed to overcome this limitation in wide-angle X-ray diffraction experiments and to extend its capability for X-ray emission spectroscopy experiments (Fig. 1 ▸). In this section, a detailed description of the newly developed instrument is provided.

### 2.1. Internal component for small-angle X-ray diffraction

**Figure 2:**
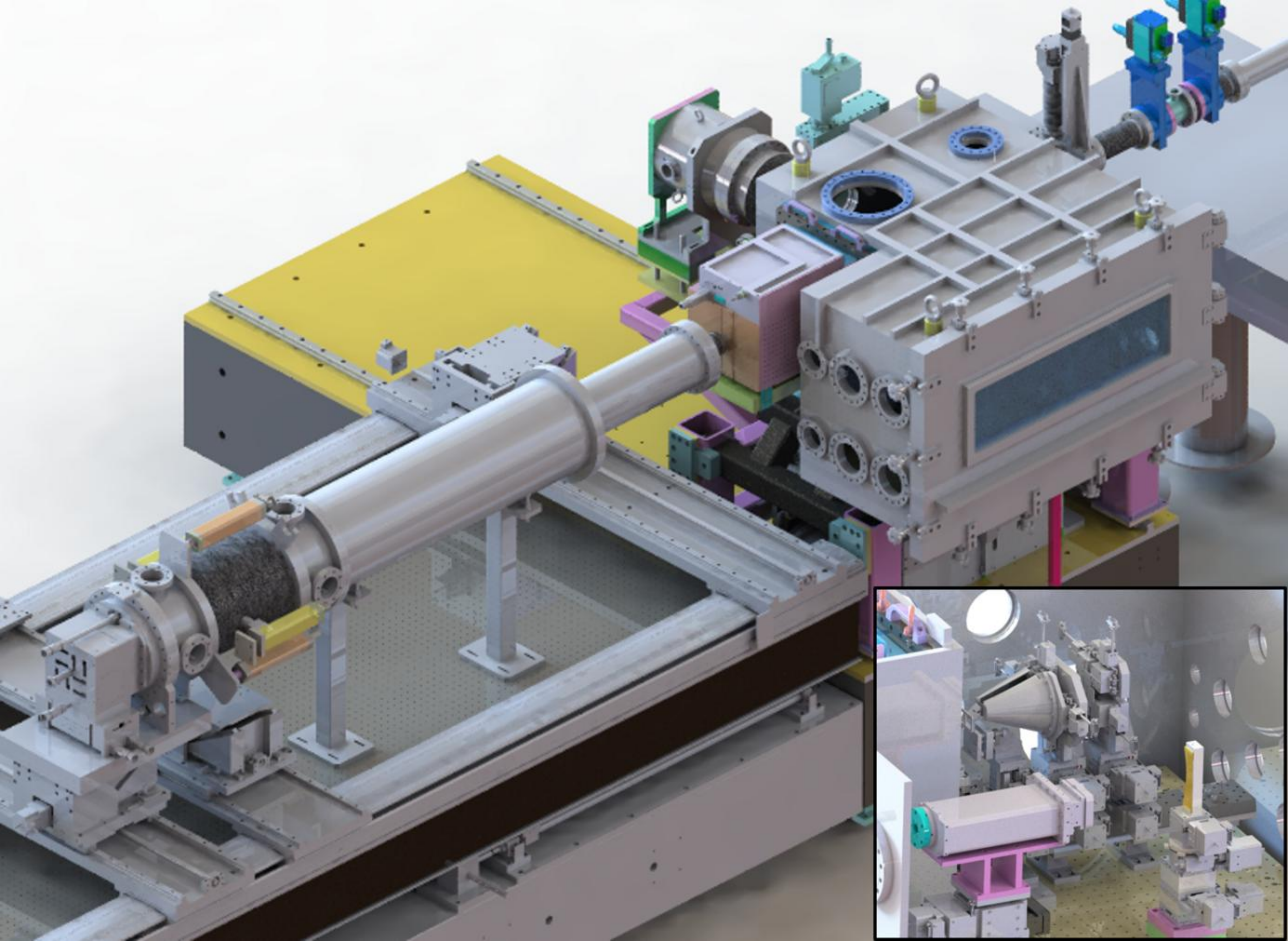
Bird’s eye-view of the multiplex imaging chamber including three JUNGFRAUs. Two detectors, JUNGFRAU 5M and JUNGFRAU 4M, are installed to collect X-ray diffraction signals. (Inset) Internal view of the multiplex imaging chamber. Two slit stages, a sample viewer stage and a sample stage are located for X-ray diffraction experiments. An in-vacuum JUNGFRAU 0.5M and a crystal stage are installed to collect X-ray emission spectra from specimens.

Fig. 2 ▸ shows the whole instrument for multiplex imaging experimentation. The multiplex imaging chamber includes two slit stages, a sample viewer stage and a sample stage designed for CDI experiments (Fig. 2 ▸). The slit is installed to block unwanted scattering from upstream optics such as X-ray focusing mirrors and make a clean background in the detection plane. The slit comprises a radiation shield with a 5 mm × 5 mm hole and four silicon blades. It is mounted on the *XZ* moving stages to allow X-rays to pass through the hole of the radiation shield. Each silicon blade with a motion stage is mounted on the radiation shield. The thickness of the blade is 0.5 mm, and its edge has a bevel cut. Downstream of the two slits, a fixed-target system with a sample viewer is positioned. The fixed-target system is embodied using stacked stages, including a fast piezo stage and raster scanning program. It is available to provide fresh samples with a repetition rate of 60 Hz, corresponding to the maximum repetition rate of PAL-XFEL. A sample viewer is located between the second slit stage and the sample stage. This is realized using a right-angle prism mirror installed in the vacuum chamber and a long-distance microscope placed outside of the chamber. The right-angle prism mirror has a 3 mm-long hole, where X-rays and the pump laser can pass through. The microscope is used to align a membrane with approximately 11 µm resolution when the magnification of the microscope is set to the maximum. The JUNGFRAU 4M is used to collect small-angle X-ray diffraction patterns of a specimen. This detector has 3.3 mm × 3.3 mm holes to pass the direct beam. The details are similar to that of a previous instrument (Sung *et al.*, 2021 ▸).

### 2.2. Detector for wide-angle X-ray diffraction

**Figure 3:**
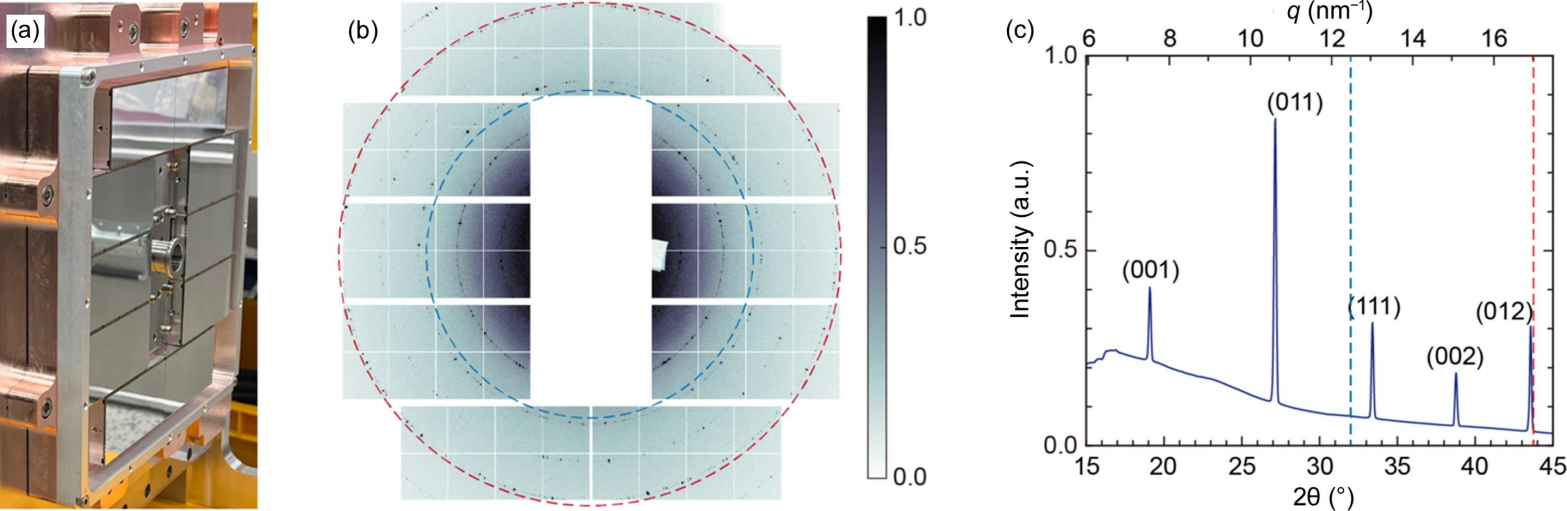
(*a*) Photograph of the JUNGFRAU 5M. Ten modular sensors are designed to collect wide-angle X-ray diffraction, and the diffracted X-rays at small angles pass through a central hole. (*b*) Single-shot diffraction pattern of LaB_6_ powder. The blue- and red-dotted lines indicate the minimum and the maximum 2θ angle covering the whole azimuthal direction, respectively. A central hole with a 20 mm diameter and dummy areas are indicated in white. (*c*) Intensity plot of the diffraction ring shown in (*b*). Debye–Scherrer rings can be measured in the range 6 nm^−1^ to 17.5 nm^−1^. Notably, whole signals of Debye–Scherrer rings spanning from 12.5 nm^−1^ to 17 nm^−1^ can be collected along the azimuthal.

A customized JUNGFRAU 5M was employed in 2023 to measure diffracted X-rays with large momentum transfer, offering atomic scale structural information of crystalline specimens [Fig. 3 ▸(*a*)]. This detector comprises ten modular sensors, and each sensor has 512 × 1024 pixels with a pixel size of 75 µm × 75 µm. The arrangement of chips is designed to record diffracted X-rays at large angles. A central hole with a diameter of 20 mm enables measurement of small-angle X-ray diffraction signals using a downstream detector without interference with other components. 25 µm-thick black Kapton is used to prevent debris and others from reaching the chips. This detector is directly connected to the multiplex imaging chamber by a customized flange. It is designed with a protruded geometry to minimize the distance between the sample and the detector.

To calibrate the sample-to-detector distance, LaB_6_ was used with 9 keV X-rays. LaB_6_ powder was uniformly spread on the polyimide film, and another film was covered to preserve the sample. Each diffraction pattern was collected from a fresh sample spot, allowing estimation of the sample-to-detector distance. Fig. 3 ▸(*b*) shows an LaB_6_ powder pattern acquired by a single X-ray pulse. The detector was positioned 106 mm away from the interaction point, and the detectable *Q* range spans from 6 nm^−1^ to 17.5 nm^−1^. Specifically, *Q*_min_ and *Q*_max_, covering the whole azimuthal direction, were ∼12.5 nm^−1^ and 17 nm^−1^, respectively. Both are indicated by blue and red dotted lines in Figs. 3 ▸(*b*) and 3 ▸(*c*). In this configuration, five peaks of LaB_6_ from the (001) peak to the (012) peak were collected [Fig. 3 ▸(*c*)].

### 2.3. X-ray emission spectrometer

**Figure 4:**
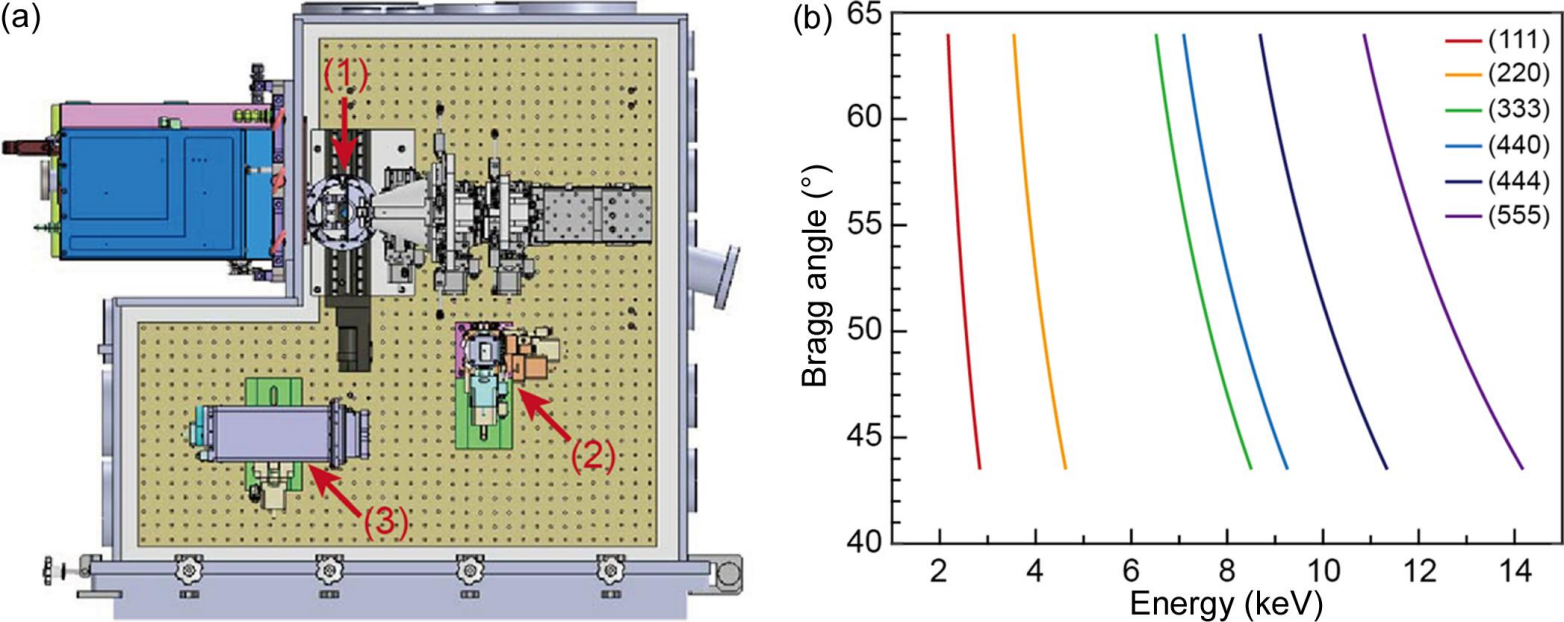
(*a*) Top view of the spectrometer installed in the multiplex imaging chamber. Three components of the von Hamos spectrometer are indicated by arrows: (1) sample stage, (2) an Si crystal analyzer with a radius of curvature of 250 mm mounted on the stacked stages, (3) JUNGFRAU 0.5M. It is located next to the sample stage. The distance between the crystal plane and the detector plane is determined by the radius of curvature of the crystal analyzer. (*b*) Energy range measured by the spectrometer with Si(111) and Si(220) crystal analyzers. Because of the finite volume of the multiplex imaging chamber, the measurable energy range is restricted.

Fig. 4 ▸(*a*) illustrates the schematic experimental setup for the von Hamos spectrometer. It enables investigation of the filled electron state of specimens. Diced Si crystals, each with dimensions of 110 mm × 30 mm × 0.3 mm, are attached to a bent silica substrate of 110 mm × 30 mm with a radius of curvature of 250 mm. There are two types of Si crystals with different indices, (111) and (110), but only one crystal can be mounted on the stacked stages. These stacked stages are composed of one manual slide, two translational motion stages and three tilting motion stages. The manual slide controls the approximate position of the crystal to meet Bragg’s law, and fine alignment is achieved using the motorized motion stage. Another translation stage is used to change the distance between the crystal and detector planes. It allows the user to focus the emitted X-rays on the detection plane. An in-vacuum type JUNGFRAU 0.5M is used as a detector to collect focused X-rays, and this detector is mounted on the combination of a manual slide and two motorized stages. The detector is aligned using these stages.

Fig. 4 ▸(*b*) shows the measurable photon energy range under this setup and each Si crystal. This spectrometer, equipped with an Si(111) or Si(220) crystal, facilitates the investigation of the specimens from tender X-rays to hard X-rays in the multiplex imaging chamber. However, the finite space of the chamber limits access to a part of the energy range using this spectrometer and crystals.

**Figure 5:**
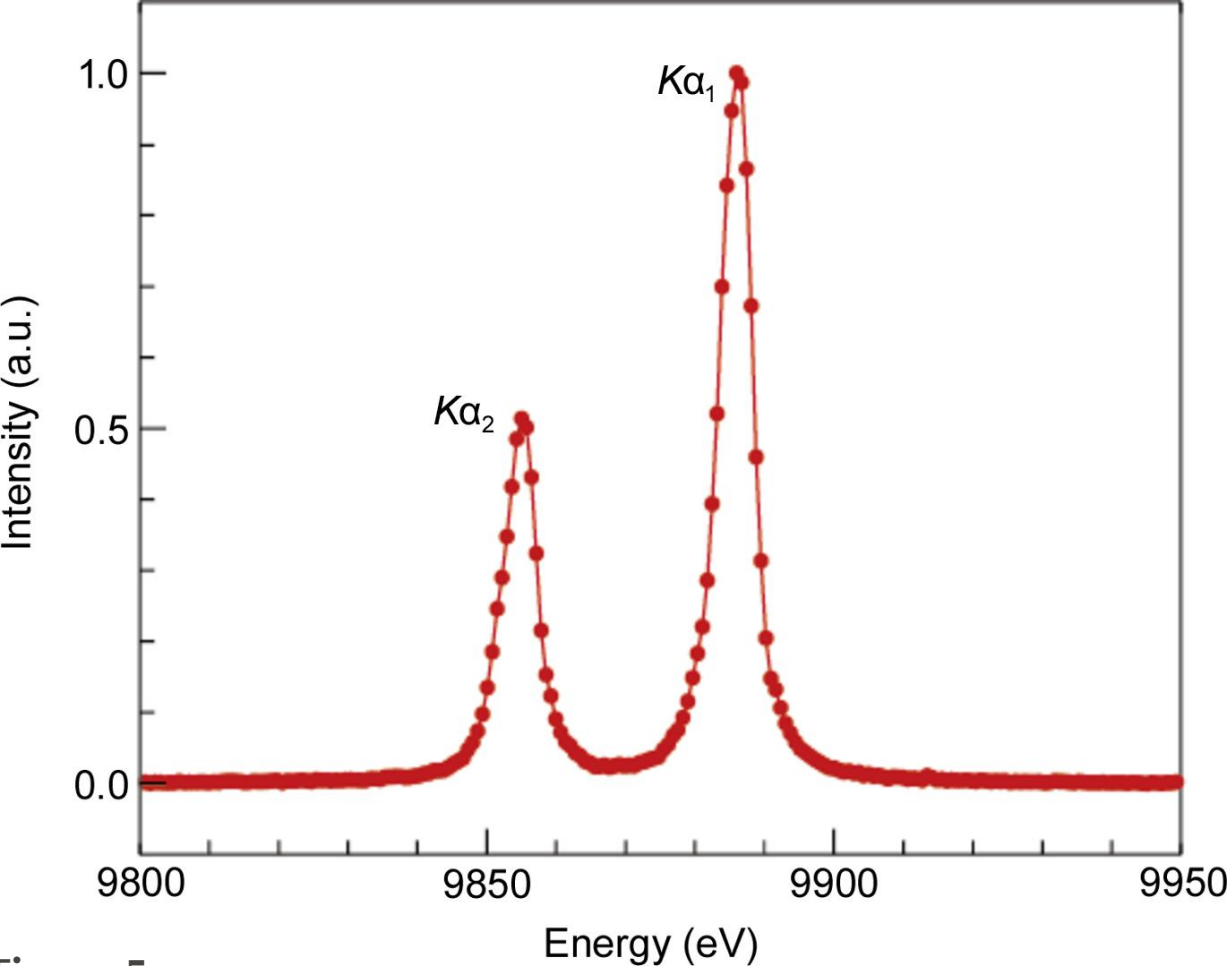
*K*α_1_ and *K*α_2_ emission lines of the Ge single crystal. Two peaks are clearly measured by the accumulation of 100 X-ray pulses.

To evaluate the performance of the spectrometer, a 20 mm × 20 mm × 0.5 mm Ge crystal was used. The incident X-ray energy was fixed at 12 keV, with a bandwidth of a few tens of electronvolts. As the focused XFEL beam makes a hole on the surface of the Ge crystal, raster scanning was used to collect Ge emission spectra from the fresh spot for each X-ray pulse. The Si(444) crystal analyzer was mounted to reflect the *K*α emission lines of Ge. Fig. 5 ▸ shows Ge *K*α_1_ and *K*α_2_ emission spectra acquired from 100 X-ray pulses. Ge *K*α_1_ and *K*α_2_ emission spectra were clearly observed by this spectrometer with the Si(444) crystal analyzer, and the peak energies of the two emission lines corresponded to 9.886 keV and 9.855 keV. The energy resolution of this spectrometer was estimated using the interval between the two emission lines as ∼0.7 eV pixel^−1^.

## 3. Experimental results and discussions

The main purpose of this newly developed chamber is to investigate samples using three X-ray techniques: small-/wide-angle X-ray diffraction and X-ray emission spectroscopy. The incident X-ray energy was determined by considering three factors: the oversampling ratio for the imaging experiment, the position of the Bragg peak under the fixed detector geometry and the X-ray absorption edge of the target atom. The results presented here demonstrate the performance of this instrument and the possibilities for further work in monitoring ultrafast phenomena. All experiments were performed using the multiplex imaging chamber at the hard X-ray beamline of PAL-XFEL.

### 3.1. Multiplex imaging experiment

We conducted multiplex imaging experiments on spherical gold nanoparticles (Au NPs) with a 100 nm diameter to show the performance of this instrument. The photon energy was considered to allow simultaneous measurements of small-/wide-angle X-ray diffraction and X-ray emission spectra, set at 12 keV, which was larger than the *L*_3_ absorption edge (11.919 keV) of Au. This allowed the detector to capture Bragg peaks of (111) and (200) from Au NPs. Au NPs were homogeneously distributed on the Si_3_N_4_ membrane using a plasma cleaner and a spin coater (Nam *et al.*, 2016 ▸). Intact Au NPs were delivered to each microfocused X-ray pulse by raster scanning.

**Figure 6:**
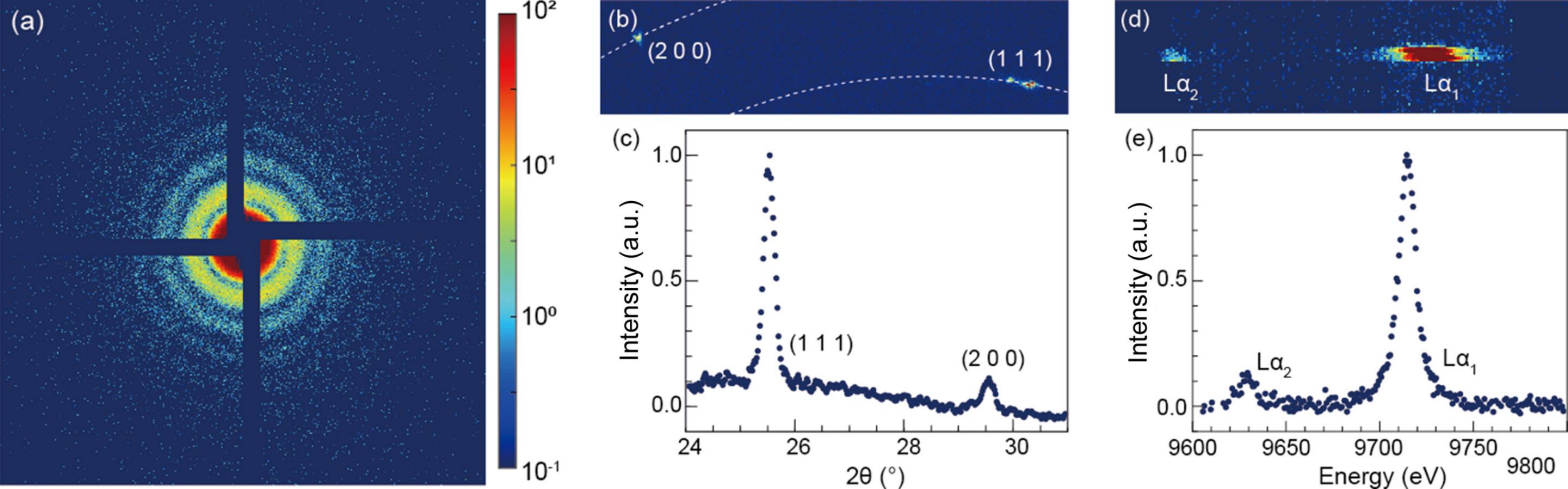
Simultaneous measurements of small-angle X-ray diffraction, wide-angle X-ray diffraction and X-ray emission spectra of Au NPs. (*a*) Small-angle diffraction pattern of a single Au NP. (*b*) Au (111) and (200) Bragg peaks from the same particle measured in (*a*). (*c*) Intensity profiles of two peaks displayed by averaging the diffraction ring azimuthally. They were normalized with the maximum intensity. (*d*) Accumulated *L*α_1_ (9.713 keV) and *L*α_2_ (9.628 keV) emission lines of the Au NPs acquired using a von Hamos spectrometer. (*e*) Intensity profiles of two emission lines. More than 1000 X-ray pulses were accumulated to improve the SNR of the emission lines.

A single-shot diffraction pattern of an Au NP was recorded, as shown in Figs. 6 ▸(*a*) and 6(*b*). In small-angle X-ray diffraction, the speckle pattern was recorded with high contrast and reflected a spherical morphology of the sample [Fig. 6 ▸(*a*)]. In wide-angle X-ray diffraction, the periodic arrangement of the sample formed strong diffraction peaks on the detector [Fig. 6 ▸(*b*)]. These observed diffraction peaks were (111) and (200), corresponding to atomic spacings of 2.35 Å and 2.04 Å, respectively. The diffraction intensity as a function of the 2θ angles is shown in Fig. 6 ▸(*c*). Using the von Hamos spectrometer with an Si(444) crystal analyzer, Au *L*α_1_ (9.713 keV) and *L*α_2_ (9.628 keV) spectra were collected. Fig. 6 ▸(*d*) clearly shows Au *L*α_1_ and *L*α_2_ spectra from an Au NP. More than 1000 X-ray pulses were exposed to enhance the signal-to-noise ratio (SNR) of the emission signal. Additionally, the bent crystal allowed the X-rays to be focused vertically, further improving SNR. The *L*α_1_ emission intensity was ∼10 times higher than the *L*α_2_ emission [Fig. 6 ▸(*e*)]. The energy resolution was 0.68 eV in this setup. Thus, the multiplex imaging instrument successfully performed simultaneous measurements on the tested Au NP samples.

### 3.2. Time-resolved multiplexing experiment using tender X-rays

The tender X-ray range included the *K*-edge absorptions of light key elements such as Na, Mg, P, S, Cl, K and Ti as well as the *L*-edge absorptions of heavier elements like Ru and Ag. Consequently, this X-ray energy range is of keen interest because of its significant promise for exploring various specimens in the fields of biology, materials science and chemistry.

**Figure 7:**
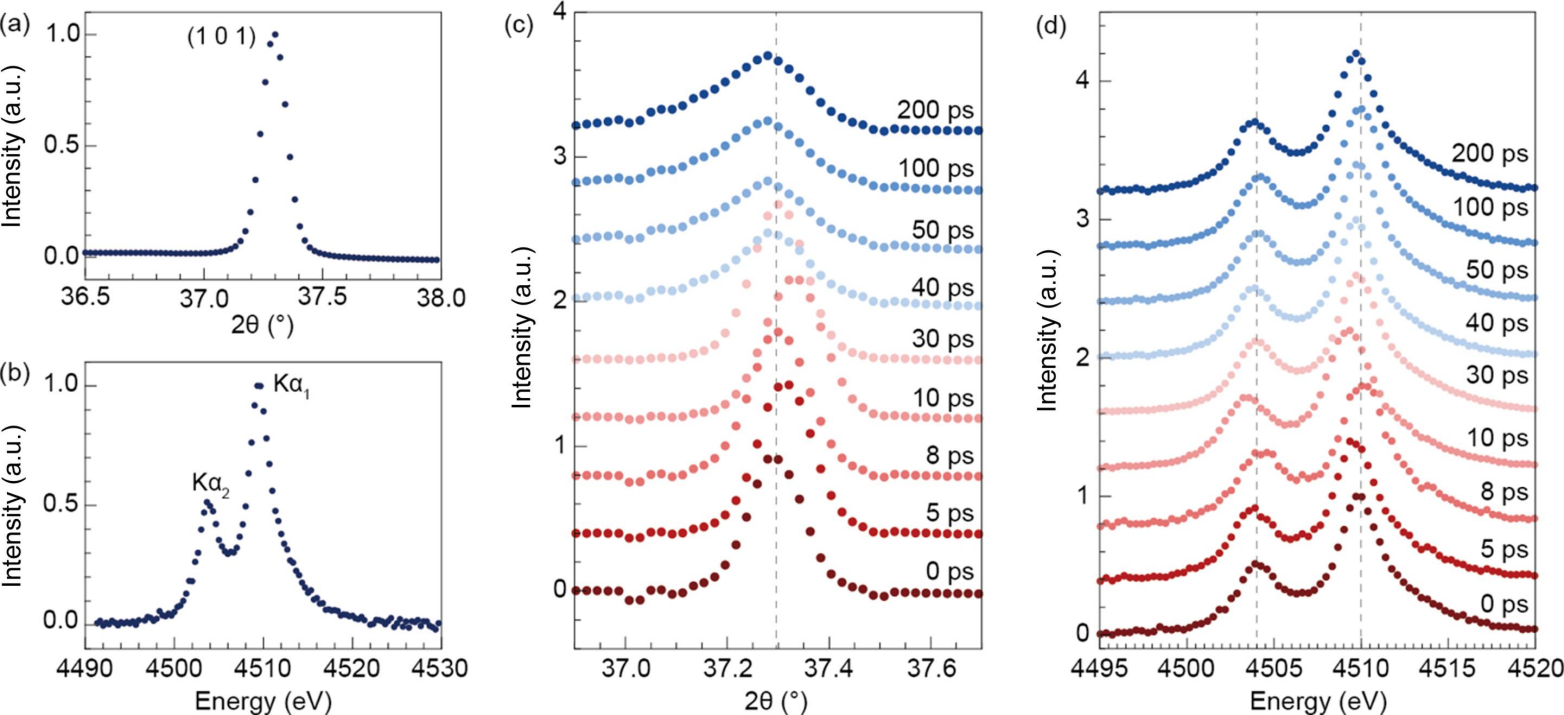
Static and time-resolved measurement of wide-angle X-ray diffraction and X-ray emission spectra of anatase TiO_2_ NPs. (*a*) Wide-angle diffraction intensity profile of the TiO_2_ (101) peak. (*b*) X-ray emission spectra of Ti *K*α_1_ (4.510 keV) and *K*α_2_ (4.504 keV). (*c*) Time-resolved X-ray diffraction of TiO_2_ NPs. At 40 ps, significant (101) peak broadening corresponding to a 70% increase in FWHM is presented. It implies melting of the crystalline domain after laser irradiation. (*d*) Time-resolved X-ray emission spectrum of TiO_2_ NPs. At 10 ps, the maximum energy shift, −0.6 eV, was observed. Compared with diffraction signals, the changes in the emission spectra were inappreciable after a 40 ps delay.

As a demonstration, we performed time-resolved multiplexing experiments using tender X-rays to follow the ultrafast phase transition of anatase TiO_2_ NPs. To enhance the transient emission signals, the sample was spread on the membrane at a high concentration. This membrane has 64 windows. The size of each window is 9 mm × 0.2 mm. This caused saturation of the JUNGFRAU 4M detector, which was used to collect small-angle X-ray diffraction data. In this experiment, only wide-angle X-ray diffraction patterns and X-ray emission spectra were recorded. The electron beam energy was adjusted to generate femtosecond X-ray pulses of 5.5 keV. The incident X-ray energy was set to be higher than the Ti *K* absorption edge. X-rays were focused to approximately 2.8 µm × 4.6 µm full width at half-maximum (FWHM) at the interaction point. For the spectrometer, an Si (220) crystal analyzer was mounted to reflect and focus Ti *K*α_1,2_ emissions only among the total fluorescence from the sample. Figs. 7 ▸(*a*) and 7 ▸(*b*) show the powder diffraction pattern of TiO_2_ (101) and the emission spectrum of Ti *K*α_1,2_ from intact TiO_2_ NPs. The energy resolution, determined by the detector pixel size, was 0.34 eV in this von Hamos geometry. In addition, diffraction patterns and emission spectra were recorded as a function of the delay time between the X-ray and pump laser. A Ti:sapphire femtosecond laser tuned to 266 nm was used as the pump source for optical excitation of TiO_2_ NPs, and the laser fluence was set to 16 mJ cm^−2^. The size of pump laser spot was ∼177 µm × 177 µm (FWHM).

A photoinduced peak shift was observed in the early time delay [Fig. 7 ▸(*c*)]. The peak shifted toward a higher angle, indicating lattice contraction of the sample, which started at a 5 ps delay and reached its maximum at a 10 ps delay. Starting from a 40 ps delay, the remarkable (101) peak-broadening corresponding to a 70% increase at FWHM was observed. This peak broadening indicates that the change in the domain size of TiO_2_ NPs could be attributed to the melting of the crystalline domain after laser irradiation. Furthermore, photoinduced changes were observed from the peak energy shift of the Ti *K*α_1,2_ emission spectrum [Fig. 7 ▸(*d*)]. The energy peak shift to lower energy was observed at a 5 ps delay, and the maximum energy shift was −0.6 eV observed at a 10 ps delay. Further, the FWHM of the peak in the emission spectrum was increased by ∼4.5% for Ti *K*α_1_ and ∼6.8% for Ti *K*α_2_ at a 10 ps delay. In contrast with diffraction signals, the changes in the emission spectra were inappreciable after a 40 ps delay. At each delay time, we exposed at least 1000 X-ray pulses. For the membrane window of 9 mm × 0.2 mm, we exposed only 25 shots to collect signals from fresh samples at different positions. The number of exposures can be determined by the size of the pump laser. In this case, we ensured a sufficient interval between each exposure to avoid any disturbance caused by the previous pump laser shot. Since we could only mount three membranes at once, we acquired a total of 4800 diffraction patterns and emission spectra through three membranes. Considering the 60 Hz operation mode of the machine and alignment procedure, it took 1.5 h. Although the experimental data had a limited time delay data point due to the finite beam time for commissioning, the multiplex imaging instrument achieved a sufficiently successful time-resolved experiment with a pump laser.

## 4. Conclusions

A new instrument was developed at the hard X-ray beamline of PAL-XFEL for multiplex imaging experiments. This instrument empowers researchers to comprehensively investigate specimens by simultaneously capturing small-angle X-ray diffraction, wide-angle X-ray diffraction and X-ray emission spectra. The morphological information and crystal arrangement of specimens were investigated using small-/wide-angle diffraction patterns. Emission spectra provide electronic structural information about a specimen. Each of these X-ray techniques contributes complementary information for comprehensive understanding of a specimen. Furthermore, we aim to extend to mounting more crystal analyzers to improve the SNR of emission signals and prepare new analyzer crystals to cover inaccessible energy ranges under this setup. These improvements should continue to advance our research capabilities.
